# Application of medical imaging methods and artificial intelligence in tissue engineering and organ-on-a-chip

**DOI:** 10.3389/fbioe.2022.985692

**Published:** 2022-09-12

**Authors:** Wanying Gao, Chunyan Wang, Qiwei Li, Xijing Zhang, Jianmin Yuan, Dianfu Li, Yu Sun, Zaozao Chen, Zhongze Gu

**Affiliations:** ^1^ State Key Laboratory of Bioelectronics, School of Biological Science and Medical Engineering, Southeast University, Nanjing, China; ^2^ State Key Laboratory of Space Medicine Fundamentals and Application, Chinese Astronaut Science Researching and Training Center, Beijing, China; ^3^ Central Research Institute, United Imaging Group, Shanghai, China; ^4^ The First Affiliated Hospital of Nanjing Medical University, Nanjing, China; ^5^ International Children’s Medical Imaging Research Laboratory, School of Biological Science and Medical Engineering, Southeast University, Nanjing, China

**Keywords:** organ-on-a-chip, tissue engineering, medical imaging, artificial intelligence, deep learning

## Abstract

Organ-on-a-chip (OOC) is a new type of biochip technology. Various types of OOC systems have been developed rapidly in the past decade and found important applications in drug screening and precision medicine. However, due to the complexity in the structure of both the chip-body itself and the engineered-tissue inside, the imaging and analysis of OOC have still been a big challenge for biomedical researchers. Considering that medical imaging is moving towards higher spatial and temporal resolution and has more applications in tissue engineering, this paper aims to review medical imaging methods, including CT, micro-CT, MRI, small animal MRI, and OCT, and introduces the application of 3D printing in tissue engineering and OOC in which medical imaging plays an important role. The achievements of medical imaging assisted tissue engineering are reviewed, and the potential applications of medical imaging in organoids and OOC are discussed. Moreover, artificial intelligence - especially deep learning - has demonstrated its excellence in the analysis of medical imaging; we will also present the application of artificial intelligence in the image analysis of 3D tissues, especially for organoids developed in novel OOC systems.

## 1 Introduction

**FIGURE 1:**
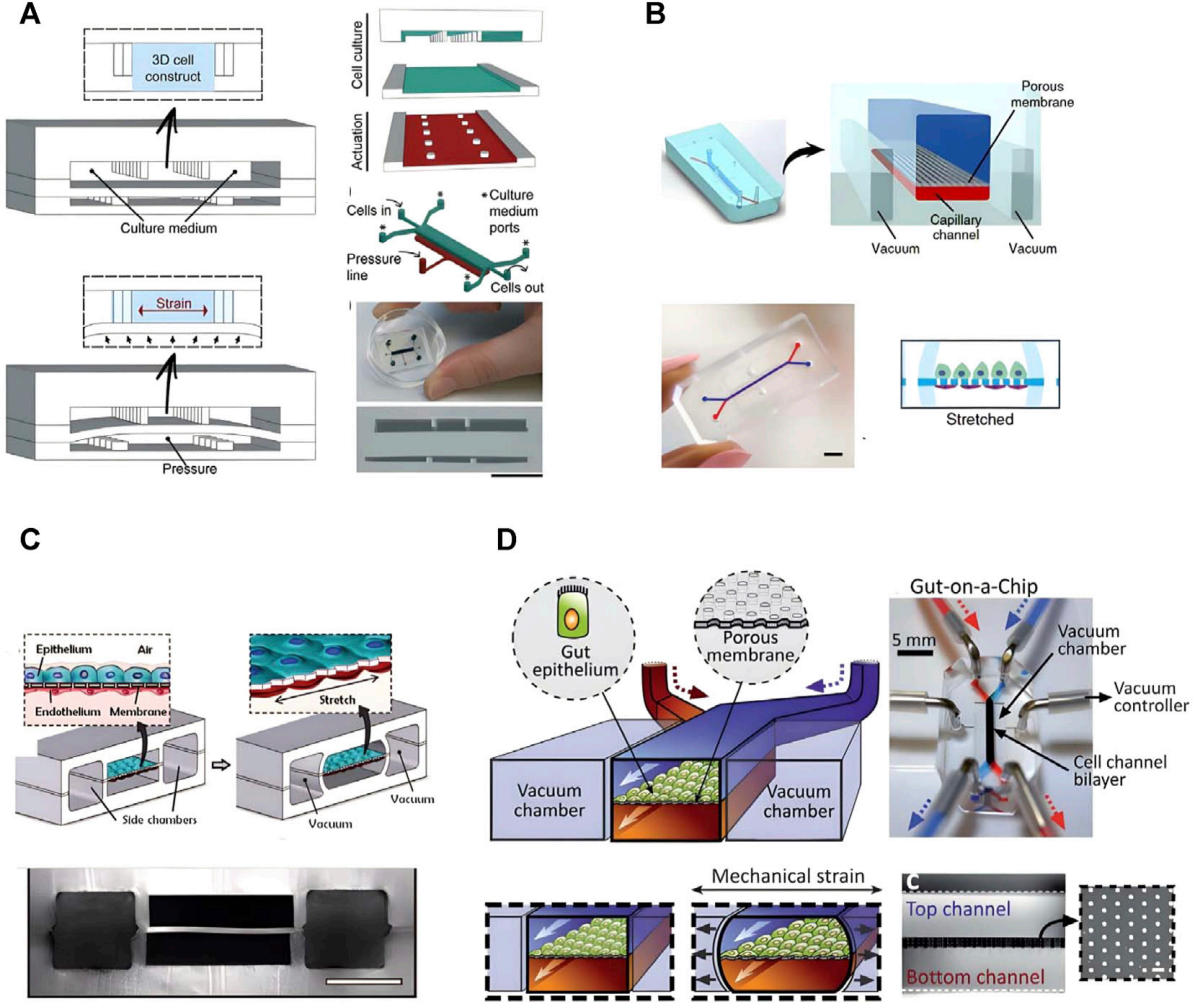
The representative chips of Organ-on-a-chip. **(A)** Heart on a chip (adapted and modified from Marsano et al., 2016). **(B)** a Glomerulus Chip (adapted and modified from Musah et al., 2018). **(C)** Lung chip (adapted and modified from Huh et al., 2010). **(D)** Intestinal chip (adapted and modified from Kim et al., 2012).

About 90% of drugs could not pass the clinical trials, even they have passed cell and animal experiments. The reason is that there are species differences between animals and humans. Thus, animals cannot accurately represent and simulate the disease status, progression and following treatment that humans have (Golebiewska et al., 2020). At the same time, the limitations of low-throughput *in vivo* animal research led to the extension of drug development life cycle and the increase of development cost. Organ-on-a-chip (OOC) is an interdisciplinary technology that combines cell biology, biomedical engineering, biomaterials, microfabrication and so on to recreate and simulate the biomedical and physical microenvironments of human organs on microfluidic chips (Wu et al., 2020; Park et al., 2019). Each unit in OOC is usually very small, so it can screen drugs with high throughput, which improves the efficiency in drug screening (Sun et al., 2019a). OOC has good potential to make up the deficiency in animal experiment, and may replace animal experiment to some extent in the future. Over the past decade, researchers have developed chips with different designs and sizes to mimic organs such as heart (Figure 1A) (Marsano et al., 2016), kidney (Figure 1B) (Musah et al., 2018), lung (Figure 1C) (Huh et al., 2010), intestine (Figure 1D) (Kim et al., 2012), and so on. OOC technology was selected as one of the top ten emerging technologies at the 2016 World Economic Forum.

**FIGURE 2:**
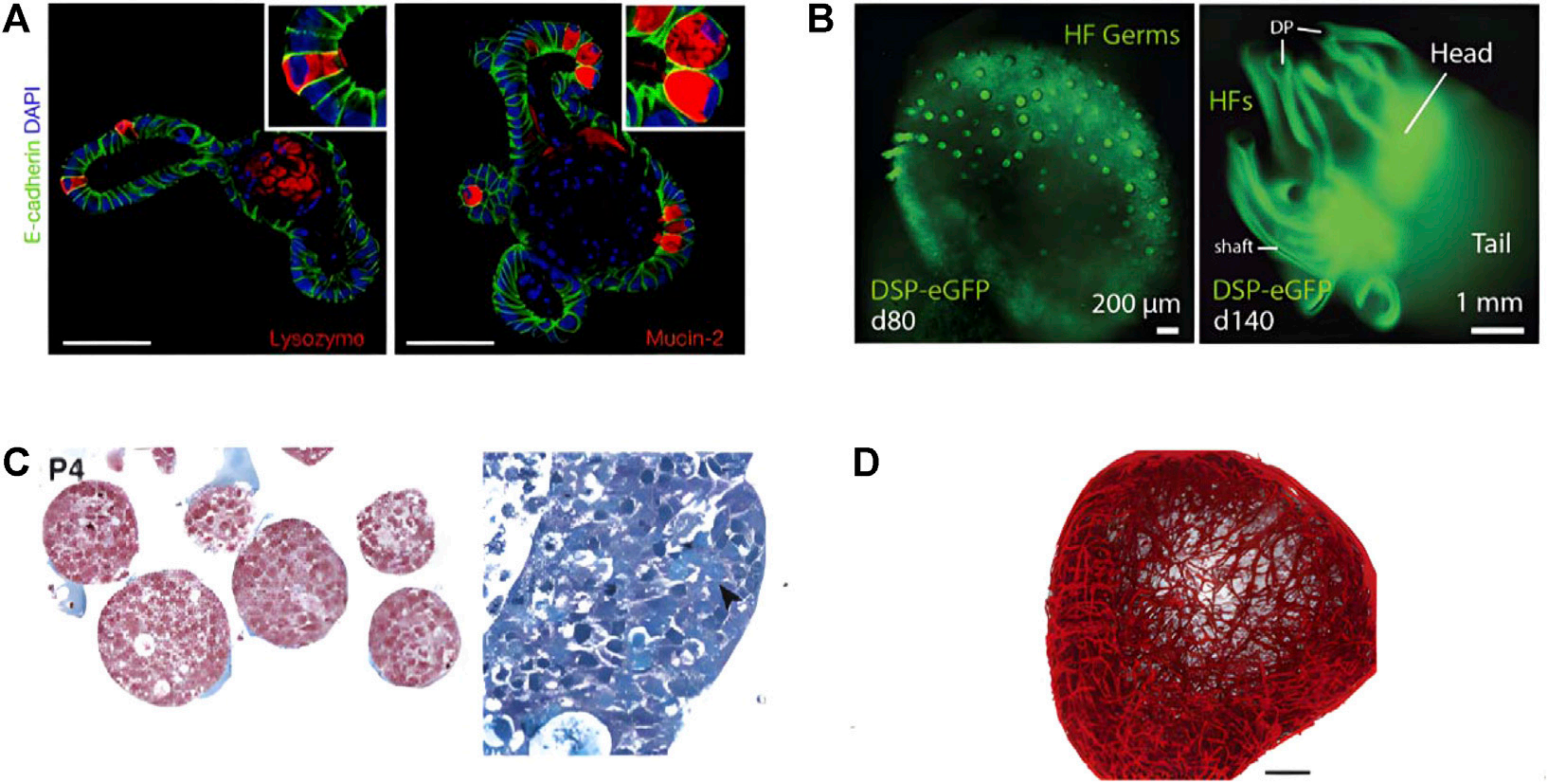
Four different types of organoids constructed by researchers. **(A)** Intestinal organoids (adapted and modified from Gjorevski et al., 2016). **(B)** Skin organoids (adapted and modified from Lee et al., 2021). **(C)** Organoid Models of Human Liver Cancers (adapted and modified from Nuciforo et al., 2018). **(D)** Human blood vessel organoids (adapted and modified from Wimmer et al., 2019).

Organoids are three-dimensional cell complexes with organ-specific functions and similar structures to organs induced and differentiated from stem cells by 3D *in vitro* culture technology (Artegiani and Clevers, 2018; Rossi et al., 2018). Organoids can be derived from induced pluripotent stem cells (iPSCs) and/or adult stem cells (ASCs) or even primary epithelial cells (Dutta et al., 2017), which are self-organized to form a three-dimensional structure that shares certain similarities to human organs. Currently, researchers have established dozens of organoids including organoids of intestine (Figure 2A) (Gjorevski et al., 2016), skin (Figure 2B) (Lee and Koehler, 2021), tumors (Figure 2C) (Nuciforo et al., 2018), blood vessels (Figure 2D) (Wimmer et al., 2019), etc. Organoids have a wide range of application values, which can be used for drug testing, understanding organ development and related diseases, promoting the research on tumor treatment, and making tissue replacement therapy possible (Lancaster and Knoblich, 2014; Bleijs et al., 2019).

While the research of organoids has made great progress, it also promotes the development of tissue engineering. The concept of tissue engineering was put forward as early as 1980. Its direct goal is to develop biological substitutes for damaged tissues or organs for clinical application. The main elements in tissue engineering include cells being seeded, supportive matrices w or w/o growth factors. The main sources of seed cells are primary tissue cells, stem cells, or progenitor cells (Berthiaume et al., 2011). Growth factors are soluble, diffusing signaling polypeptides that regulate different kinds of cell growth processes (Bakhshandeh et al., 2017). The activity and compatibility of biomaterials are also constantly improving to help regulate cell proliferation, migration, differentiation, and other behaviors (Khademhosseini and Langer, 2016). Tissue engineering has practical applications in the fields of skin replacement and cartilage repair, and significant progress has also been made in the fields of blood vessels, liver, and spinal cord (Langer and Vacanti, 2016). Researchers have already used organoid technology for *in vitro* tissue construction. Markou et al. use vascular organoids derived from human pluripotent stem cell derived mural cell phenotypes for tissue engineering (Markou et al., 2020). Reid et al. use organoids and 3D printing for consistent, reproducible culture of large-scale 3D breast structures (Reid et al., 2018). Organoid technology is expected to become a platform for tissue engineering in the future.

Though OOC and organoid have been developed and widely used in recent biological and biomedical sciences, the analyzing methodology of these models are still very limited and old-fashioned. Researchers often use very traditional paraffin-embedding with sectioning and/or cryo-sectioning to analyze slices of those tissues, while these operations are high in labor-requirement and low in efficacy. It is difficult to collect three-dimensional images due to their high in thickness and poor in light transmittance; thus, imaging with traditional light microscopy could not reach tissues in depth while having decent spatial resolution. While in the tissue engineering technology that complements and develops with organoids, medical imaging methods have been widely used and have great reference significance. Therefore, this article will review the medical imaging methods that may be used in organoid and OOC imaging, including CT/microCT, MRI/small animal MRI, OCT, etc. We will overview the pros and cons of different medical imaging methodologies, focusing on spatial resolution and image contrast analysis; the unique setup for medical imaging instruments and its applications in organoid imaging will need to be explored and specified. This article will also review the application of 3D printing combined with medical imaging technology in tissue engineering and OOC technology.

Finally, we will discuss the applications of artificial intelligence (AI) in different medical imaging methods and the image analysis of organoids, including detecting and tracking organoids, predicting the differentiation of organoids, and so on (Kegeles et al., 2020; Bian et al., 2021). The main methods reviewed in this article are mainly machine learning in artificial intelligence, especially deep learning. Most deep learning models are based on artificial neural networks (Gore, 2020). The artificial neural network is an algorithm inspired by human brain neuron cells, aiming to simulate the way the human brain processes problems. Therefore, deep learning is essentially a neural network with three or more layers. Deep learning can be widely used in speech recognition, image recognition, natural language processing, and other fields. At present, artificial intelligence has made significant progress in the field of medical imaging. Artificial intelligence can help provide critical diagnostic information, improve image reading efficiency, and reduce the inevitable errors of human image reading. Specific functions include but are not limited to image quality improvement, lesion detection, automatic segmentation, classification, quantification, etc. (Currie et al., 2019; Higaki et al., 2019; Zhao and Li, 2020).

## 2 High spatial resolution imaging method

### 2.1 Overview

The spatial resolution and some other properties of five medical imaging tools are listed and compared in Table 1. Each instrument has different temporal and spatial resolutions, and the corresponding use scenarios are also different.

**TABLE 1:** Properties of different medical imaging methods.

	Spatial resolution	Temporal resolution	Soft Tissue contrast	Penetration depth	Scan time	Cost
MRI	0.5–5 mm	2–50 ms	High	Full body	Long	High
Small animal MRI	5–200 μm	20–50 ms	High	Full body	Long	High
CT	0.5–0.625 mm	60–200 ms	Low	Full body	Short	Low
Micro-CT	5 μm	50 ms	Low	Full body	Short	Medium
OCT	10–15 μm	20–50μs	Medium	About 2 mm	Short	Low

Adapted from Refs. (Lin and Alessio, 2009a; Lin and Alessio, 2009b; Cao et al., 2009; Runge, 2009; McCabe and Croce, 2012; Nam et al., 2015a; Lewis et al., 2016; Zbontar et al., 2018; Aumann et al., 2019)

MRI, magnetic resonance imaging; CT, computed tomography; OCT, optical coherence tomography.

### 2.2 Magnetic resonance imaging

Magnetic resonance imaging (MRI) is an important non-invasive imaging method for medical diagnosis based on the principle of nuclear magnetic resonance (Hespel and Cole, 2018). Protons precession in a strong magnetic field. When the frequency of the electromagnetic wave emitted to the proton is equal to the precession frequency, the proton will resonate and produce a transition. When the external energy pulse disappears, the proton will return from the ordered high-energy state to the disordered low-energy state and release radio waves, which can be received by the receiving coil and fall into the radio frequency range. The released energy follows the exponential decay form (Yousaf et al., 2018). The time used to release energy is called relaxation time. The relaxation time of different biological tissues is different, which is also the core principle of nuclear magnetic resonance imaging. The field strength of MRI equipment used in the clinic is mainly 1.5T and 3T. Equipment with higher field strength has a higher signal-to-noise ratio and contrast. The uMR Jupiter 5.0T has been developed for clinical whole-body scanning imaging. It shows better image quality and performance in detecting tiny details in various organs, as well as provides more precise quantitative analysis (Zhang et al., 2022). MRI is often used in the brain, blood vessels, spinal cord, abdominal and pelvic organs, musculoskeletal and so on, which can be used to study brain tumors, Parkinson’s disease, mental diseases, and so on (Meijer and Goraj, 2014; Liu et al., 2020a). MRI signal needs spatial positioning (Hamilton et al., 2017), which takes longer time compared with other imaging methods. Still, it will not cause damage to human body or imaging tissue due to the use of non-ionizing electromagnetic radiation. Perfusion MRI was studied to evaluate perfusion parameters at the capillary level. It can be divided into two categories: using and not using exogenetic contrast agents (Jahng et al., 2014). Magnetic resonance spectroscopy (MRS) is a non-invasive metabolic imaging technology based on the same principle as MRI. MRS is most commonly obtained by ^1^H. In addition, it can also be obtained by ^13^C, ^31^P, and other nuclei (Speyer and Baleja, 2021). Single voxel spectroscopy (SVS) is the most commonly used and easily obtained MRS technology (Zhang et al., 2018), which is limited to receiving signals from a single voxel. Multi-voxel chemical shift imaging (CSI) techniques, including 2D and 3D CSI, have a larger coverage area, which can be displayed as a single spectrum, a spectral map, or a color metabolic image (Zoccatelli et al., 2013). MRS can be used to study the metabolic changes of Alzheimer’s disease, amyotrophic lateral sclerosis, brain tumor disease, etc.

MRI has also been used in organoid research. Vascular organoids are imaged to observe whether vascular tissue functions normally. The researchers construct organoid-based orthotopic mouse xenograft models, transplant the endometrial cancer organoid cultured *in vitro* into the mouse uterus, and observe the tumor growth every week with MRI (Espedal et al., 2021). Researchers have also proposed the possibility of using MRI to study brain organoids (Badai et al., 2020).

#### 2.2.1 Small animal magnetic resonance imaging

In the process of translational research and drug development, animal models are needed for research. It is necessary to perform brain imaging of rodents, mostly rats or mice, to observe the phenotypic characteristics of the disease in order to help understand the mechanism of mental illness, especially in the research of neurological diseases (Herrmann et al., 2012; Hoyer et al., 2014). The brain structure of the animal model is tiny, reaching the sub-millimeter level (Gao et al., 2019), and the reduction of voxel volume will lead to a reduction in the signal-to-noise ratio (Boretius et al., 2009). The images produced by the human scanner are not clear on the details of the mouse brain. These demands lead to the study of high-resolution MRI for small animal imaging. Some researchers optimize T2 weighted fast spin echo MRI at 9.4 T to realize the imaging of mouse brain cell layer (Boretius et al., 2009). At present, many manufacturers have developed instruments specially used for MRI imaging of small animals. Compared with human scanners, they have higher spatial and temporal resolution, requiring the use of strong magnets, special gradient coils, and the development of special sequences for small animals (Jakob, 2011). There are also many researchers who are committed to transforming human scanners to image small animals. Some studies connect preclinical magnets and gradient coils to human scanners, making it possible to achieve high-resolution imaging (Felder et al., 2017); a surface loop array is proposed to image small animals on human scanners (Gao et al., 2019).

### 2.3 Computed tomography

Computed tomography (CT) is a commonly used medical image diagnosis method in clinics. It measures the attenuation of x-beams in different projection layers of the human body and finally carries out mathematical reconstruction by computer to synthesize it into three-dimensional images (du Plessis et al., 2018). The initial CT used a translational scanning system. With the advancement of technology, CT scan has gradually evolved into fan beam scanning, electron beam scanning, etc. The number of rows of detectors in CT scans is increasing, and the scanning time is getting shorter and shorter. At present, multi-slice spiral CT scans, such as 64-slice spiral CT have become the mainstream of the market because of their fast-imaging speed and clear imaging. In addition to ordinary scans, CT can also perform enhanced scans by injecting contrast agents to make the lesions appear more clearly. Lung, heart, and blood vessels are suitable for CT examination (Wiant et al., 2009; Cox and Lynch, 2015; Thillai et al., 2021).

#### 2.3.1 Micro-computed tomography

Micro-computed tomography (micro-CT) is a cone-beam computed tomography scanning technology. The principle is the same as that of clinical CT, both of which are x-ray attenuation imaging. The difference is that the critical structure is a micro-focus x-ray tube and a high-resolution x-ray detector. Micro-CT can perform *in vitro*, *in vivo*, and *ex vivo* studies and is an essential method for preclinical imaging (Bartos, 2018). With the deepening of research, the spatial resolution of micro-CT has been continuously improved, and the imaging field of view has been reduced. Therefore, micro-CT has been applied in the fields of histomorphological analysis, bone quality assessment, small animal imaging, 3D printing and other fields that require more precision (Orhan, 2020). It enables nondestructive visualization of specimens in 2D and 3D. Tan et al. use micro-CT to analyze the microstructure of mouse calvarial bone (Tan et al., 2022). Doost et al. use iodine-enhanced micro-CT to image the mouse heart *ex vivo* (Doost et al., 2020).

### 2.4 Optical coherence tomography

Optical coherence tomography (OCT) is a non-invasive, high-resolution optical imaging technique that distinguishes different tissues by analyzing the difference between the incident signal and the received signal, taking advantage of the different degrees of absorption and scattering of light by different tissues (Podoleanu, 2005; Podoleanu, 2012). OCT is mainly composed of a low coherence light source, Michelson interferometer and photoelectric detection system. According to different signal acquisition units, it can be divided into time domain OCT (TD-OCT) and frequency domain OCT (FD-OCT) (Chaber et al., 2010; Mueller et al., 2010). TD-OCT developed earlier, using a mechanical reference mirror. FD-OCT improves the imaging speed and sensitivity, accelerates the development of OCT, and has become the mainstream of application. FD-OCT can be realized by spectra-domain OCT (SD-OCT) and swept-source OCT (SS-OCT) (Podoleanu, 2012). The spatial resolution of OCT is high, up to several microns, but due to the insufficient penetration of light into the tissue, the imaging depth is between 1 and 3 mm (McCabe and Croce, 2012). Therefore, OCT is suitable for precision medical fields such as intravascular imaging and ophthalmic diseases (Kim et al., 2017). In the field of intravascular imaging, the application scenarios of OCT basically overlap with that of IVUS, but it can provide more detailed intracoronary pathological features (McCabe and Croce, 2012). At the same time, OCT can also be used to evaluate bioabsorbable vascular stents (Okamura et al., 2010; Brugaletta et al., 2012). In the field of ophthalmic diseases, OCT has become the primary imaging method. The initial imaging of the posterior end such as the retina and the optic nerve head, has progressing to the imaging of the anterior segment such as the ocular surface and the anterior segment due to the development of FD-OCT (Fu et al., 2017; Bille, 2019). The development of OCT greatly promotes the research of glaucoma, macular degeneration and other ophthalmic diseases and plays a great auxiliary role in the research of some diseases that may cause retinopathy, such as Alzheimer’s disease and Parkinson’s disease (Cheung et al., 2015; Zou et al., 2020). Compared with CT, MRI, and other imaging technologies commonly used in the clinic, OCT has higher spatial resolution, and higher imaging depth compared with confocal microscope and other microscopic imaging technologies. Therefore, the emergence of OCT makes up for the gap between traditional medical imaging technology and microscopic imaging technology and can provide support for the biomedical field of organoids with thicker tissue.

## 3 3D Printing in tissue engineering and organ-on-a-chip

3D printing and 3D bioprinting technology have introduced tissue engineering and OOC technology as a standardized culture platform, which also requires medical imaging support.

### 3.1 3D printing and 3D bioprinting

3D printing has made considerable progress in recent years. 3D printing creates three-dimensional objects by superimposing layers on a two-dimensional plane, which is versatile and customizable. 3D printing has been applied and improved in aerospace, manufacturing, and so on. When 3D printing is combined with medicine, it has evolved further. More and more researchers in the field of biomedical engineering take 3D printing as a transformation tool for biomedical applications. The slice data of medical images can be modeled and printed layer by layer through 3D printing to visualize simulated organs or other structures. This helps researchers study pathology, helps students learn biological structures, and helps patients better understand their own diseases.

3D bioprinting is an application of 3D printing in biomedicine and has become a promising method for tissue engineering and regenerative medicine. Compared with 3D printing, 3D bioprinting uses living cells, biological materials, etc. as “bioinks” to construct artificial multicellular tissues or organs in three dimensions (Dey and Ozbolat, 2020). It can be used to manufacture a three-dimensional framework that has a similar hierarchical structure to living tissues. Currently, popular 3D bioprinting technologies include laser-assisted bioprinting, inkjet bioprinting, and micro-extrusion bioprinting (Zhu et al., 2016). There have been 3D bioprinting studies on skin, bones, liver, nerves, blood vessels, etc. It is expected to produce transplantable biological tissues in the future to meet the demand for organ transplantation (Matai et al., 2020).

**FIGURE 3:**
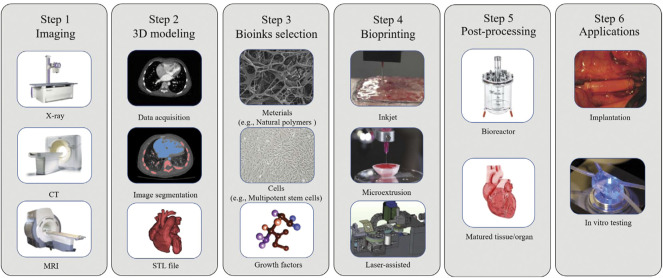
A typical process of 3D bioprinting includes 6 steps: 3D modeling, bioink selection, bioprinting, post-processing and application. (adapted and modified from Murthy et al., 2014; Vijayavenkataraman et al., 2018; Lee et al., 2021).

Figure 3 shows a typical process of 3D bioprinting; the main steps are imaging, 3D modeling, bioinks selection, bioprinting, post-processing, and applications. It can be seen that there is a close relationship between 3D bioprinting and medical imaging. The first step of 3D bioprinting is to image the tissue or organ to be printed through medical imaging equipment such as CT and MRI (Abdullah and Reed, 2018). The second step is that 3D modeling depends on accurate image segmentation (Squelch, 2018), which can be supported by artificial intelligence. In the final stage of application, medical images can also be used to visually inspect the tissues *in vitro* or transplanted into the body.

### 3.2 3D printing in tissue engineering

Conventional tissue engineering strategies employ a “top-down” approach in which cells are seeded on biodegradable polymer scaffolds (Nichol and Khademhosseini, 2009), but these approaches often fail to distribute cells rationally and provide a microenvironment for cell survival. The bottom-up modular approach has the advantage of assembling microenvironments, which is more conducive to constructing large-scale biological tissues (Mandrycky et al., 2016). Therefore, 3D printing has brought new impetus to the development of tissue engineering. 3D printing can be used in tissue engineering to rationally assemble multiple types of cells and scaffold materials for tissues. There are already impressive results using 3D printing to build skin, cartilage, blood vessels, etc. for tissue engineering. 3D printing in tissue engineering can be divided into scaffolded and scaffoldless methods. There has been tremendous progress in 3D printing methods with scaffolds. 3D printing can print precise and complex scaffolds for tissue engineering, and it is convenient to introduce computer methods to assist scaffold construction (Zaszczyńska et al., 2021). There are already 3D printed scaffolds for tissue engineering using materials such as metals, ceramics, hydrogels, etc. Wu et al. achieve 3D printing of microvascular networks using a hydrogel layer (Wu et al., 2011). Lee et al. use polycaprolactone (PCL) to create a framework for hepatocyte engineering (Lee et al., 2016). Scaffold-free approach exploits self-assembly processes in developmental biology (Richards et al., 2013). Taniguchi et al. use 3D bioprinting technology to construct a scaffold free trachea with spheroids composed of several types of cells (Taniguchi et al., 2018). Lui et al. demonstrate the enhancement of mechanical stimulation by creating scaffold-free heart tissue from hiPSC-derived cardiomyocyte spheroids (Lui et al., 2021).

### 3.3 3D printing in organ-on-a-chip

3D printing has also been applied in the field of OOC. The microfluidic device of OOC is mainly made by using traditional manufacturing techniques. The more complex the organizational structure, the more complex and time-consuming the microfluidic device of OOC is. Since 3D printing has the advantage that complex spatial structures can be freely designed, it can change the method of fabricating microfluidic devices (Carvalho et al., 2021). The microfluidic device constructed by 3D printing has the characteristics of high accuracy and short time from design to manufacturing (Goldstein et al., 2021). Sochol et al. investigate the potential of using 3D printing to make Kidney-on-a-Chip platforms (Sochol et al., 2016). The liver chip developed by Lee et al. Using 3D printing significantly enhance liver function (Lee and Cho, 2016). The advantages of 3D printing, which is easy to design and implement, will break the technical barriers that exist in the multidisciplinary intersection of OOC, and accelerate the development and innovation of OOC (Knowlton et al., 2016).

## 4 Application of medical imaging in tissue engineering and artificial tissues

**FIGURE 4:**
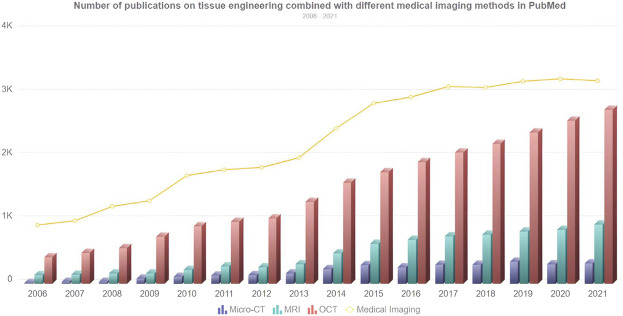
Number of publications on tissue engineering combined with different medical imaging methods in PubMed. The line chart represents the overall trend of the number of searches for medical imaging keywords, and the bar chart represents the number of searches for micro-CT, MRI, and OCT from 2006 to 2021.

With the development of tissue engineering, the composition of artificial tissue has become increasingly complex, and advanced imaging techniques are required to evaluate the structure of the tissue (Nam et al., 2015b). The micro-CT, MRI, and OCT imaging techniques reviewed above can be applied to artificial tissue imaging. These advanced imaging techniques enable nondestructive visualization studies compared to some traditional tissue engineering techniques that may destroy the sample. Figure 4 shows the development trend of the number of publications combining tissue engineering and various medical imaging methods from 2006 to 2021. It can be seen that the number of publications is growing steadily, whether it is medical imaging keyword search or different medical imaging methods.

### 4.1 Magnetic resonance imaging in tissue engineering

**FIGURE 5:**
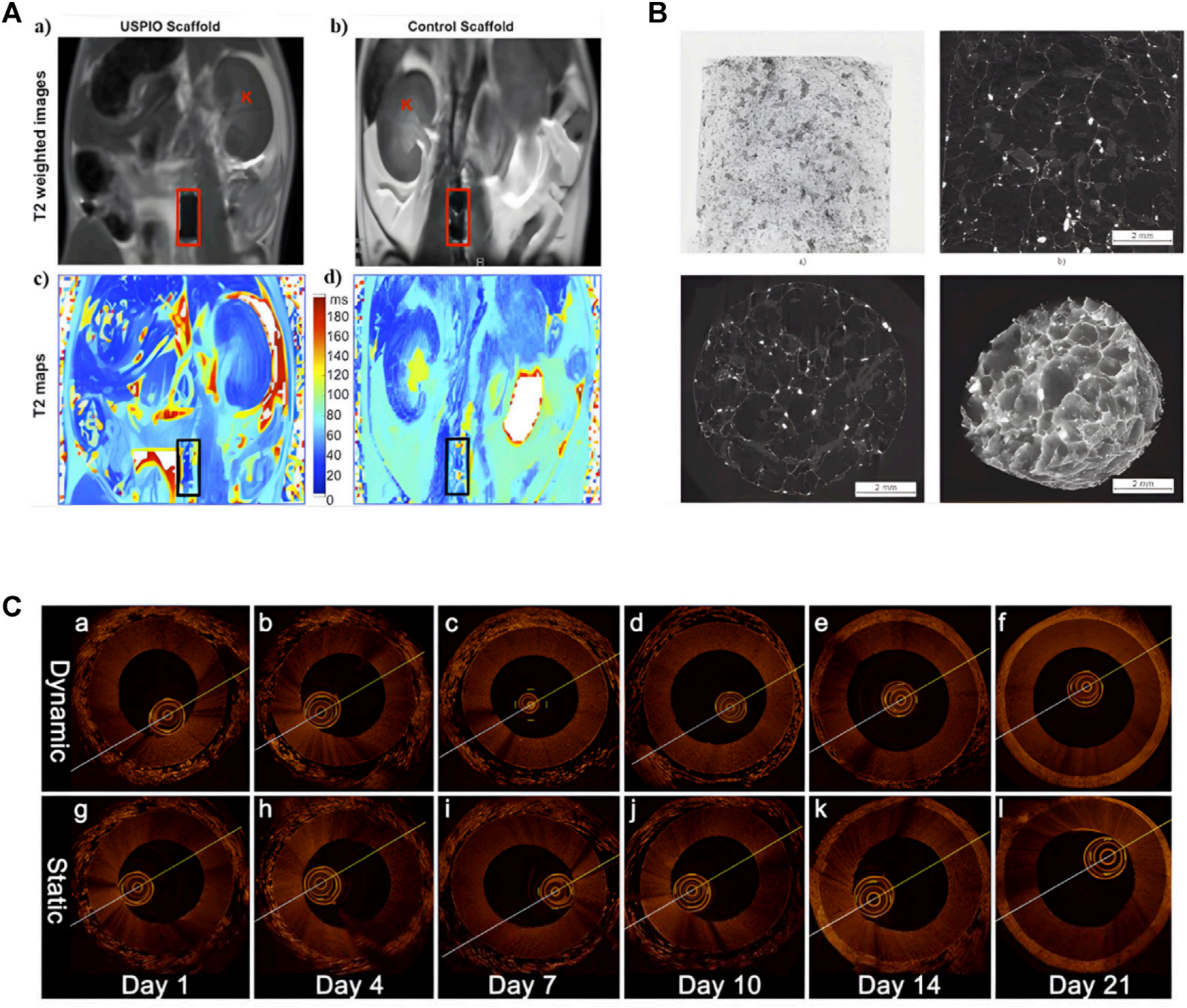
Application of Different Imaging Methods in Tissue Engineering. **(A)** Noninvasive MRI images of labeled and unlabeled stent-grafts in mice, a, b) RARE T2-weighted images of labeled (a) and unlabeled. (b) seed scaffolds after implantation. Boxes represents the location of the graft and K represents kidneys. c, d) Corresponding T2 maps of a, b) (adapted and modified from Harrington et al., 2011). **(B)** micro-CT scanning of collagen-based scaffold (adapted and modified from Bartoš et al., 2018). **(C)** OCT imaging contrasting the effects of pulsatile stimulation on tissue-engineered vascular grafts culture, (a–f) are images with arterial stimulation, (g–l) are images without arterial stimulation (adapted and modified from Chen et al., 2017).

MRI can image artificial tissue implanted in the body. Fujihara et al. use MRI to evaluate the maturity of cartilage tissue transplanted into the back of mice (Fujihara et al., 2016). Using small-animal MRI tracking imaging in experimental mice, Apelgren et al. demonstrate that gridded 3D bioprinted tissue allows vascular ingrowth after implantation. Harrington et al. use cellular MRI to continuously image the grafted tissue of artificial blood vessels, realizing the serial study of MRI at the cellular level of tissue engineering (Figure 5A) (Harrington et al., 2011). MRI is also an important tool for imaging tissue engineering scaffolds. Szulc et al. synthesize MnPNH2 for labeling of dECM scaffolds and visualize the scaffolds using MRI, demonstrating the potential for long-term detection of dECM-based tissue engineering (Szulc et al., 2020). Marie et al. use high-resolution 1.5-T MRI to evaluate scaffold structure and detect cell seeding (Poirier-Quinot et al., 2010). Using gadolinium-enhanced MRI to measure negative fixed-charge density in tissue-engineered cartilage *in vitro*, Miyata et al. assess its relationship to biomechanical properties (Miyata et al., 2010).

### 4.2 Micro-computed tomography in tissue engineering

The use of micro-CT in tissue engineering has increased significantly, especially in imaging hard tissue. Martin et al. apply micro-CT to tissue engineering scaffolds aimed at bone regeneration, assessing structural changes related to hydration, complementing traditional methods that can only be studied in the dry state (Figure 5B) (Bartoš, 2018). Tim et al. model the bone tissue engineering scaffold based on micro-CT images to evaluate the structural performance (Van Cleynenbreugel et al., 2006). Wang et al. use MICROFIL perfusion and micro-CT for 3D reconstruction of rat blood vessels, helping to analyze the number, diameter, connectivity and other parameters of blood vessels as an objective assessment method for the generation of angiogenesis in tissue-engineered nerves (Wang et al., 2016). Cioffi et al. use micro-CT to construct a 3D model of a cartilage scaffold to help quantify the regulation of cartilage growth by hydrodynamic shearing (Cioffi et al., 2006). Townsend et al. use it to image tracheal tissue engineering to quantify tracheal patency for standardization in future production (Townsend et al., 2020). In addition, Papantoniou et al. use contrast-enhanced nanofocus CT for full-structure imaging of tissue engineering, which has great potential in 3D imaging and quality assessment of tissue engineering (Papantoniou et al., 2014).

### 4.3 Optical coherence tomography in tissue engineering

OCT is also used in tissue engineering and is especially suitable for imaging tissues with collagen matrix in tissue engineering, such as skin and tendons. Smith et al. use SS-OCT to monitor dermal rehealing of cutaneous wounds (Smith et al., 2010). Yang et al. use PS-OCT to image tissue-engineered tendon (Yang et al., 2010). Chen et al. demonstrate the effect of pulsatile stimulation on the development of engineered blood vessels using OCT for real-time imaging of tissue-engineered vascular grafts (Figure 5C) (Chen et al., 2017). Yang et al. monitor the cell contour and polylactic acid scaffold in tissue engineering by OCT (Yang et al., 2005). Levitz et al. assess the influence of atherosclerotic plaque composition on morphological features of OCT images (Levitz et al., 2007). Ishii et al. use two imaging techniques, OCT and magnetic resonance angiography to assess the patency of tissue engineered biotubes (Ishii et al., 2016). From the above, micro-CT, MRI, and OCT are developing continuously in tissue engineering and artificial tissue imaging, and are increasingly used by researchers.

## 5 Application of medical imaging in organoids and organ-on-a-chip

Medical imaging technology has played an important role in the construction of tissue-engineered artificial tissue. With the development and maturity of OOC technology, medical imaging technology also has the opportunity to become an imaging analysis method for OOC.

### 5.1 Application of medical imaging in organoids

**FIGURE 6:**
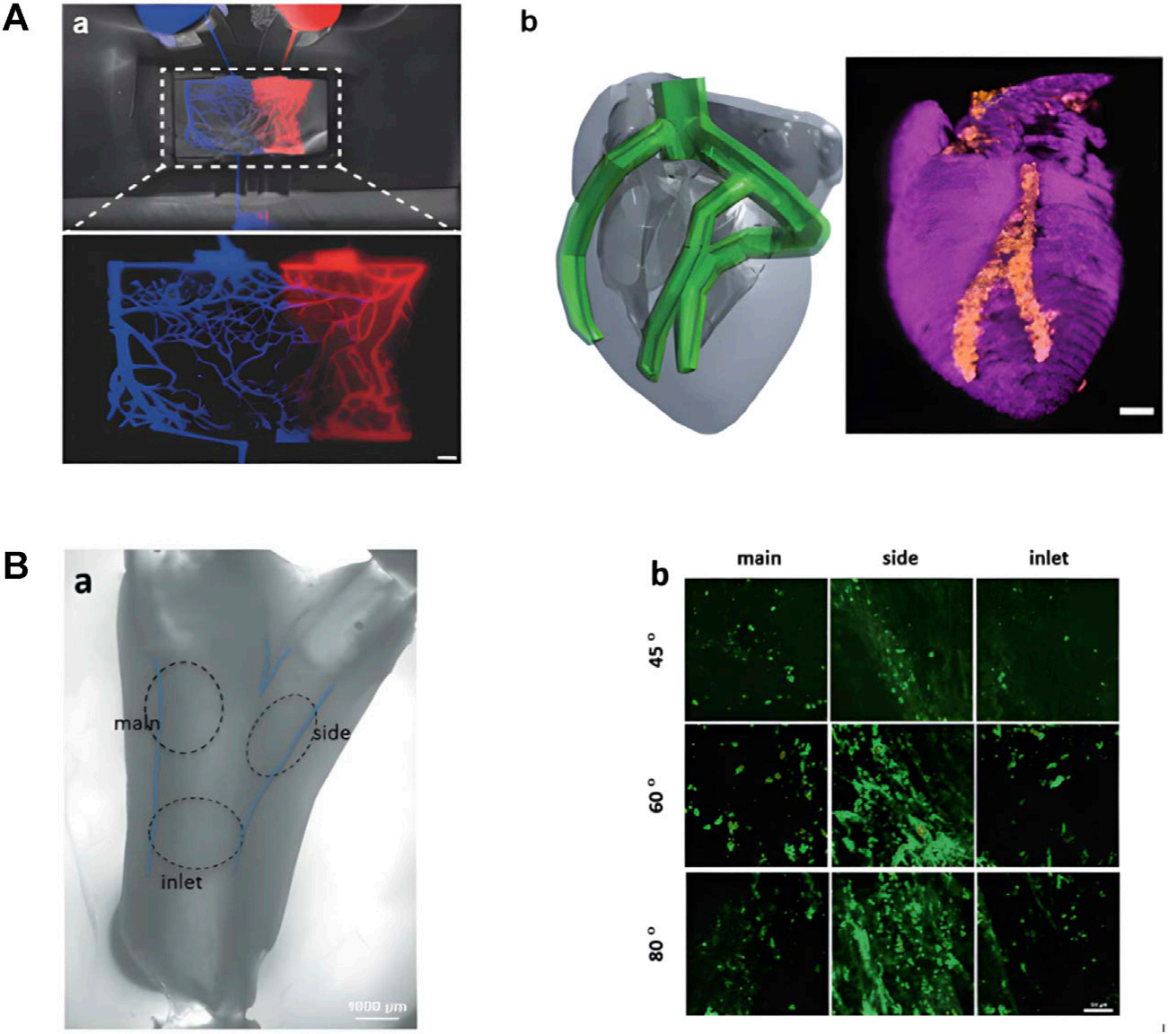
Research on artificial tissue with potential application in medical imaging. **(A)** Related research on artificial microvascular system. (a)Microvascular Networks Using Laser Patterns in Polyethylene Glycol Hydrogels. (b) 3D printed heart perfusion model (adapted and modified from Fleischer et al., 2020). **(B)** Brightfield and fluorescence images of brTEBV. (a) Brightfield image of brTEBV with a branch angle of 45° considering MC adhesion, where the dashed circles mark the inlet, side, main regions. (b) Fluorescence images of green-labeled MCs in the brTEBV region (adapted and modified from Lee et al., 2021).

Among the imaging methods reviewed, MRI has powerful functions and strong soft tissue contrast, which can be applied to most organoid imaging. Perfusion MRI may help provide perfusion parameters of the complex capillary network in artificial microvascular systems currently under study (Figure 6A) (Fleischer et al., 2020). The soft tissue contrast of CT or micro-CT is not as good as that of MRI, and is suitable for positional imaging of tissue that differs in density, such as imaging tumor organoids in tissue with altered density or size. The spatial resolution of OCT is high, but the contrast of soft tissue is relatively general. It is mainly used for eye, skin, and intravascular imaging in clinical practice. The same application field also serves as a reference for the application of OCT in organoids. Lee et al. construct branched tissue-engineered blood vessels to mimic early atherosclerosis (Figure 6B) (Lee et al., 2021). Skin organoids are also emerging as human models for dermatological research (Figure 2B) (Lee and Koehler, 2021). OCT is expected to play an important role in the research of artificial blood vessels and skin organoids.

### 5.2 Application of medical imaging in organ-on-a-chip

When using medical imaging to image OOC, it is vital to consider not only the characteristics of the organoid but also the microfluidic chip. Magnetic metals have an adverse effect on MRI, so when MRI or small animal MRI is required for OOC, metal components should be avoided early in microfluidic chip design and during processing. Additionally, if imaging with micro-CT or CT, additional consideration should be given to possible artifacts caused by microfluidic chips, resulting in problems such as image distortion. To remove artifacts, the structure of the OOC can be skillfully laid out in the early stage, and appropriate algorithms including artificial intelligence algorithms can be used in the later stage. It is foreseeable that the application of medical imaging on OOC is not only involved in one of the links but needed to be considered comprehensively and added to the entire design process. The introduction of medical imaging into the field of OOC will help OOC to industrialize and perform large-scale imaging examinations in the future.

## 6 AI achievements in medical imaging and organoids

### 6.1 Magnetic resonance imaging combined with artificial intelligence

**FIGURE 7:**
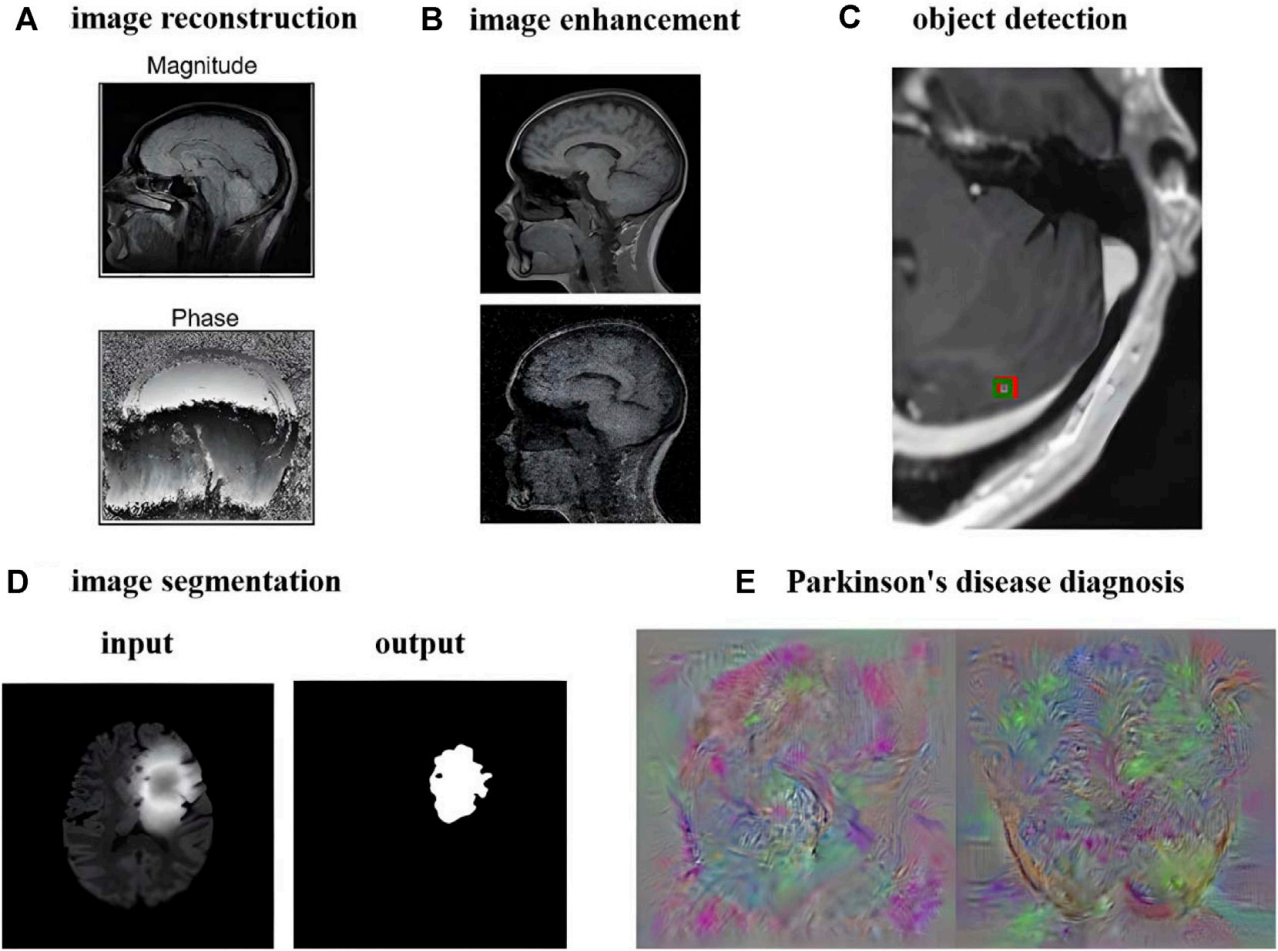
Deep Learning Image Processing and Analysis Using Brain MRI as an Example. **(A)** Image reconstruction of brain MRI (adapted and modified from Lundervold et al., 2019). **(B)** Image denoising of brain MRI (adapted and modified from Lehtinen et al., 2018). **(C)** Smallest brain metastasis detected by artificial intelligence method marked with red bounding box (adapted and modified from Zhang et al., 2020). **(D)** Brain Tumor Segmentation Using UNet++ (adapted and modified from Zhou et al., 2020). **(E)** Feature images extracted by Parkinson’s diagnostic network (adapted and modified from Sivaranjini et al., 2020).

MRI has good soft tissue contrast, so the research and analysis of MRI images are very extensive and multifaceted. Research on MRI images has evolved from traditional methods to artificial intelligence methods. This paper mainly reviews the aspects of image reconstruction, image enhancement, object detection, image segmentation, diagnosis and prediction in the order of processing and analysis of MRI. Figure 7 takes brain MRI as an example to show the current research results of artificial intelligence methods.

#### 6.1.1 Image reconstruction

**FIGURE 8:**
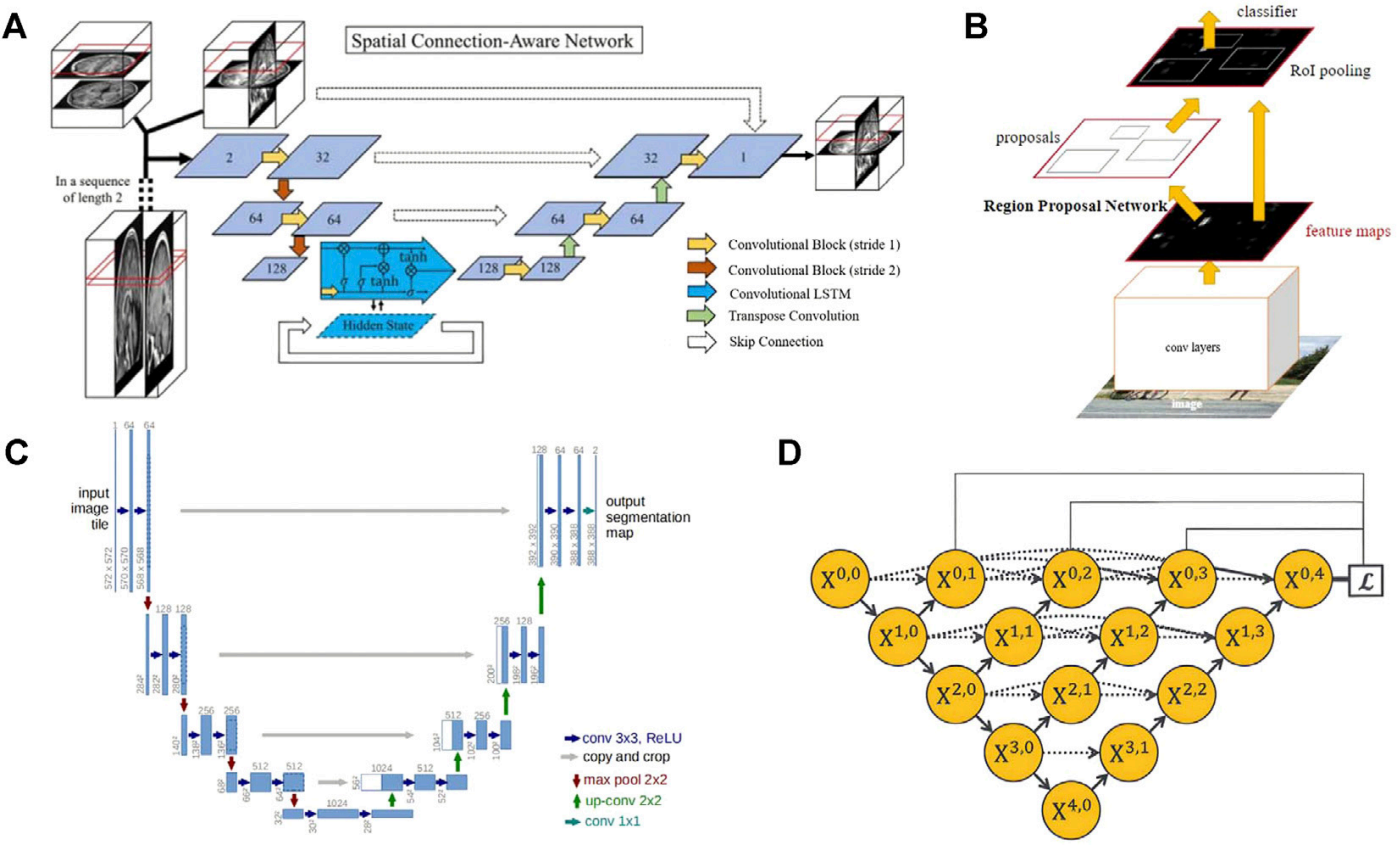
Some network frameworks applied in MRI image processing and analysis. **(A)** Spatial connectivity-aware network including LSTM blocks, exploiting sagittal information from adjacent slices. (adapted and modified from Li et al., 2019). **(B)** The Faster R-CNN network structure has two branches, the bounding box regression network and the classification network. The region proposal network is used to recommend bounding boxes that may have targets. (adapted and modified from Ren et al., 2015). **(C)** U-net is often used as a basic network. The blue boxes represent feature maps with different number of channels, the white boxes represent the copied feature maps, and the arrows represent operations such as convolution, pooling, etc. (adapted and modified from Ronneberger et al., 2015). **(D)** The UNet++ network obtained by improving U-Net, the downward arrow indicates downsampling, the upward arrow indicates down adoption, and the dot arrow indicates skip connections between feature maps. (adapted and modified from Zhou et al., 2019).

The use of deep learning for image reconstruction is a relatively new field compared to the detection and segmentation of medical images, but it has shown better performance than traditional iterative, compressed sensing and other methods in the accelerated reconstruction of images and the improvement of reconstruction quality (Lundervold and Lundervold, 2019). The long scanning time can be an issue limiting the application of MRI in organoid researches, which may be solved by some acceleration methods like half Fourier imaging, parallel imaging and compressed sensing. However, the acceleration of these methods is quite limited and the image quality always suffers from the introduced reconstruction artifacts. As a potential alternative, AI-assisted compressed sensing (ACS) integrate the mentioned techniques and innovatively introduce the state-of-art deep learning neural network as the AI module into the reconstruction procedure, which lead to a superior image quality under a high acceleration factor with fewer artifacts (Wang et al., 2021a). Schlemper et al. use cascaded CNN to reconstruct the under-sampled two-dimensional cardiac MRI. It has the function of iterative algorithm to remove aliases, and it is less prone to overfitting than a single CNN network (Schlemper et al., 2018). The experimental result can reach 11 times of under-sampling, and the entire dynamic sequence can be reconstructed within 10s. Hammernik et al. propose a variational network as a variational model, which uses deep learning to learn all free parameters to accelerate MRI image reconstruction. Finally, the reconstruction time on a single graphics card is 193 ms, showing fast computing performance (Hammernik et al., 2018). Huang et al. introduce motion information into unsupervised deep learning model for dynamic MRI reconstruction for the first time (Huang et al., 2021). Kamlesh et al. combine the domain knowledge of traditional parallel imaging with U-Net for MRI reconstruction, and the reconstruction results are anti-interference and accurate (Pawar et al., 2021). Hossam et al. use a complex valued revolution network, which uses U-Net as the backbone network to join the complex valued revolution, etc. to accelerate the reconstruction of highly undersampled MRI (El-Rewaidy et al., 2020). Li et al. use 3D U-Net to construct the brain structure and adopted the recurrent convolutional network embedding LSTM to complete more detailed vector information depiction in two steps, which retains the important features of brain MRI (Figure 8A) (Li et al., 2021a). Image reconstruction is developing rapidly. Many artificial intelligence methods that combine traditional methods or directly use deep learning to complete rapid or even real-time reconstruction are still being proposed.

#### 6.1.2 Image enhancement (de-noising, super-resolution)

Image de-noising and image super-resolution have become important research directions to improve the quality of MRI images, especially after the introduction of deep learning into this field. Despite the continuous development and innovation of medical imaging equipment, it is still inevitable to generate random noise, which will affect the speed and accuracy of doctors’ judgment. Most denoising methods are based on a small range of homogeneous samples. Benou et al. study the denoising problem of dynamic contrast-enhanced MRI and construct an ensemble of expert DNNs to train different parts of the input image separately (Benou et al., 2017). Li et al. use a supervised learning network constructed by two sub-networks to learn distribution information in MRI to reduce Rician noise (Li et al., 2020a). Noise2noise, an unsupervised learning method, has also attracted widespread attention. It is characterized by the fact that the input and output of the network are all noisy pictures during training, which is very suitable for scenes such as MRI, where it is difficult to obtain clean samples. Some researchers also propose a denoising method based on a large range of multidisciplinary samples. Sharif et al. combine attention mechanism and residual learning modified by noise gate to build deep learning network, applied to radiology, microscopy, dermatology medical images (Sharif et al., 2020). The final results show good denoising effect, which also proposes a new idea for medical image denoising.

Directly generating high-resolution images using medical imaging devices is sometimes a time-consuming and expensive process, so researchers have attempted to use deep learning to perform super-resolution operation on the images for post-processing. Neonatal brain MRI and cardiac MRI images are two important application scenarios of super-resolution. Low resolution (LR) image training samples are usually obtained by downsampling the collected images. Masutani et al. build 4 CNNs to demonstrate the excellent performance of deep learning for super-resolution of Cardiac MRI images, which is expected to shorten the scanning time for image acquisition and reduce the discomfort of patients holding their breath for too long (Masutani et al., 2020). Generative Adversarial Networks (GAN) can speed up the training time, so many researchers use GAN combined with CNN to build a training network. Based on GAN, Delannoy et al. take differential low resolution (LR) images as input and simultaneously complete two tasks of neonatal brain MRI super-resolution and segmentation (Delannoy et al., 2020). Chen et al. also implement MRI super-resolution based on GAN. The generator part of GAN uses a self-designed multi-level densely connected network (Chen et al., 2018). Zhao et al. perform parallel filtering of the original images to obtain LR images as a training set, use enhanced deep residual networks for single image super-resolution, and make different training distinctions between 2D and 3D MRI images (Zhao et al., 2020). Some researchers have also realized the joint processing of image denoising and super-resolution. Gao et al. study the super-resolution and denoising of flow MRI. They introduce physical information into the network and realize the training of the network without high-resolution labels (Gao et al., 2021).

### 6.1.3 Object detection

Object detection is an important link in medical image processing, usually using a square frame to mark and locate areas of interest such as lesions and organs, which is a preprocessing step for further segmentation or classification. Especially for small target lesions, locking the location of the lesions in advance and storing only the surrounding areas for semantic segmentation are conducive to reducing storage consumption and improving the accuracy of segmentation (Kern and Mastmeyer, 2021). It can be divided into detection of 2D MRI slices and 3D MRI image sets. The object detection of 2D images is to feed each slice of the MRI into the training network separately, which can obtain more training data and correspondingly more training volume than 3D object detection (Kern and Mastmeyer, 2021). But the disadvantage is that the context information will be lost. The current development trend is 3D object detection, which can make more full use of context information to improve the detection accuracy. In the research field of independent detection of 2D MRI, Zhou et al. use the transfer learning method and a special similarity function to pre-train the CNN in the pre-prepared data, and realize the use of unlabeled images of the lumbar spine to detect the vertebral position during the training process (Zhou et al., 2018). Zhang et al. use the classic Faster-RCNN (Figure 8B) to detect brain cancer metastasis, which has superior performance and application prospects (Zhang et al., 2020a). In the field of 3D detection, Alkadi et al. use 3D sliding window for prostate cancer detection (Alkadi et al., 2018). Qi et al. use a 3D CNN to detect cerebral microbleeds (CMBs), achieving a high sensitivity of 93.16% (Qi et al., 2016). Mohammed et al. use YOLO and 3D CNN to detect CMBs.

### 6.1.4 Image segmentation

Image segmentation aims to describe the contour of organs, tissue structures, and lesions as accurately as possible or identify the voxel set in them. Since MRI is good at depicting human soft tissues, especially the human brain, segmentation of MRI has attracted great interest from researchers. Meanwhile, the noise and artifacts of MRI images have brought challenges to segmentation (Despotović et al., 2015). Fully Convolutional Network (FCN) is the pioneer of currently popular medical image segmentation based on convolutional neural networks (CNN) (Shelhamer et al., 2017). In the multi-atlas and diffeomorphism-based encoding block, both MRI intensity profiles and expert priors from deformed atlases were encoded and fed to the proposed FCN. The MRI intensity and the expert priors from the deformation map are coded and input, and the adaptive size patches are used at the same time (Wu and Tang, 2021). The Mask RCNN framework also has a good performance in medical image segmentation. Zhang et al. use Mask RCNN to achieve tumor segmentation for breast MRI, achieving an accuracy of 0.75 on the test set (Zhang et al., 2020b) The U-Net architecture proposed by ronneberger et al. has the structure of u-encoder and decoder and the unique skip connection to help compensate for the information loss in the down sampling process (Figure 8C) (Ronneberger et al., U-Net). The design performs well in medical image segmentation and is widely used by researchers as the basic network for research. V-Net expands the segmentation method of U-Net from two-dimensional images to three-dimensional images. It uses a new loss function called dice coefficient, and replaces the pooling layer in the U-Net architecture with a convolutional layer to achieve fast (1s) and accurate (approximately 87%) volume segmentation of prostate MRI images (Milletari et al., V-Net). UNet++ (Figure 8D) is a collection of U-Net with different depths and redesigned the skip-connection in U-Net. Segmentation experiments are carried out on 6 different biomedical images, including 2D and 3D applications for brain tumor MRI image segmentation (Zhou et al., 2020). The proposal of nnUnet verify the rationality of the original U-Net framework. It only needs to adaptively set the data fingerprint and pipeline fingerprint according to different tasks. The result has won the 2020 MRI-based BraTS brain tumor segmentation competition (Isensee et al., 2021). At the same time, some researchers use recurrent neural networks (RNN) for segmentation. Rudra et al. use recurrent fully-convolutional network (RFCN) for multi-layer MRI cardiac segmentation. Recurrent networks can help extract context information from adjacent slices and improve segmentation quality (Poudel, Lamata, Montana). Andermatt et al. use RNN with multi-dimensional gated recurrent units to segment a brain MRI data set, showing a powerful segmentation ability (Andermatt et al., 2016). Transformer has begun to be applied to the field of medical image segmentation. Peiris et al. propose a U-shaped transformer architecture similar to U-Net, which specially designed the self-attention layer of the encoder and decoder, and showed promising results in MRI brain tumor segmentation (Lehtinen et al., 2018).

#### 6.1.5 Diagnosis and prediction

With the improvement of computing level, computer-aided diagnosis has become the development trend in clinical medicine, but since decision-making must be very cautious, it also requires high accuracy. MRI-based deep learning methods have been widely experimented and studied by researchers, and have been applied to disease diagnosis on MRI images of the brain, prostate, breast, kidney, etc. disease diagnosis can be regarded as a classification problem in neural networks, including distinguishing diseased and non-diseased patients, and subdividing the disease of diseased patients. Among them, the diagnosis of diseases based on brain MRI is the most abundant. Sivaranjini et al. use AlexNet and transfer learning network to classify Parkinson’s disease patient population and refine Parkinson’s disease diagnosis (Sivaranjini and Sujatha, 2020). There are also researchers apply to the diagnosis of multiple sclerosis (MS) (Shoeibi et al., 2021), Alzheimer’s disease (Roy et al., 2019), identifying schizophrenia (Oh et al., 2020). Liu et al. classify prostate cancer based on 3D multi parameter MRI (Liu et al., 2017). Gravina et al. use transfer learning combined with traditional radiology experience three time points to diagnose breast cancer lesions with dynamic contrast enhanced MRI (Gravina et al., 2019). Shehata et al. create an early diagnosis of acute renal transplant rejection diagnostic system based on diffusion-weighted MRI, which can realize a fully automatic detection process from renal tissue segmentation to sample classification (Shehata et al., 2016).

Prediction of physical development and disease progression is also a hot area. Amoroso et al. use multiplex networks to accurately assess brain age (Amoroso et al., 2019). Markus et al. use CNN and tree based machine learning methods to evaluate the age of young people based on 3D knee MRI (Mauer et al., 2021). Adrian et al. use parallel convolution paths and inception networks to predict the disease progression of MS (Tousignant et al., 2019). Li et al. use an ensemble of three different 3D CNNs for survival prediction of brain tumors based on multimodal MRI (Sun et al., 2019b). There are also studies to predict the progression of Alzheimer’s disease (Jo et al., 2019), amyotrophic lateral sclerosis survival prediction (van der Burgh et al., 2017), etc.

### 6.2 Computed tomography combined with artificial intelligence

CT and MRI are both primary imaging methods in radiology, and the problems to be solved by artificial intelligence are similar. In terms of image reconstruction, Tobias et al. map the filtered back projection algorithm to a neural network to solve the problem of limited-angle tomography, and introduce cone-beam back-projection to overcome the defect that back projection cannot complete the end-to-end network during CT reconstruction (Wurfl et al., 2018). Solomon et al. evaluate a commercial deep learning reconstruction algorithm, and the noise is greatly reduced compared to traditional methods (Solomon et al., 2020). In terms of image enhancement, low-dose CT is often used in order to reduce radiation damage to the human body, but it is accompanied by a reduction in image quality. Li et al. use improved GAN with the Wasserstein distance and a hybrid loss function including sharpness loss, adversarial loss, perceptual loss, etc. for low-dose CT image denoising (Li et al., 2021b). At the same time, low-dose CT also has the problem that the deep learning method is difficult to generalize due to different doses. In order to solve this problem, Shan et al. propose a transfer learning network cascaded by five identical networks, which does not need to be retrained with different doses (Shan et al., 2019). Yao et al. improve convolutional layers and introduce edge detection layers for denoising of micro-CT (Yao et al., 2021). To obtain high-resolution CT images, Zhao et al. construct a network with superior performance using multi-scale attention with multiple branches and information distillation (Zhao et al., 2021). Zhang et al. design a lightweight GAN, construct dense links in all residual blocks in the generator and introduce Wasserstein distance in the loss function to achieve super-resolution (Zhang et al., 2021). In the field of CT image detection, Holbrook et al. use CNN to detect lung nodules in mice based on micro-CT (Holbrook et al., 2021). Lee et al. use three CNN models for coronary artery calcium detection based on CT images. Similarly, the application of image segmentation algorithms in lung CT is more comprehensive, including lung segmentation (Yahyatabar et al., 2020), lung lobe segmentation (Gu et al., 2021), lung parenchyma segmentation (Yoo et al., 2021), lung nodule segmentation (Jain et al., 2021) and other segmentation from larger organs to smaller lesions. Shah et al. test the performance of different deep learning models for COVID-19 detection based on CT images, among which VGG-19 performs the best (Shah et al., 2021). Chen et al. construct a deep learning model with asymmetric convolution based on CT images for predicting the survival rate of non-small cell lung cancer (Chen et al., 2021a).

**FIGURE 9:**
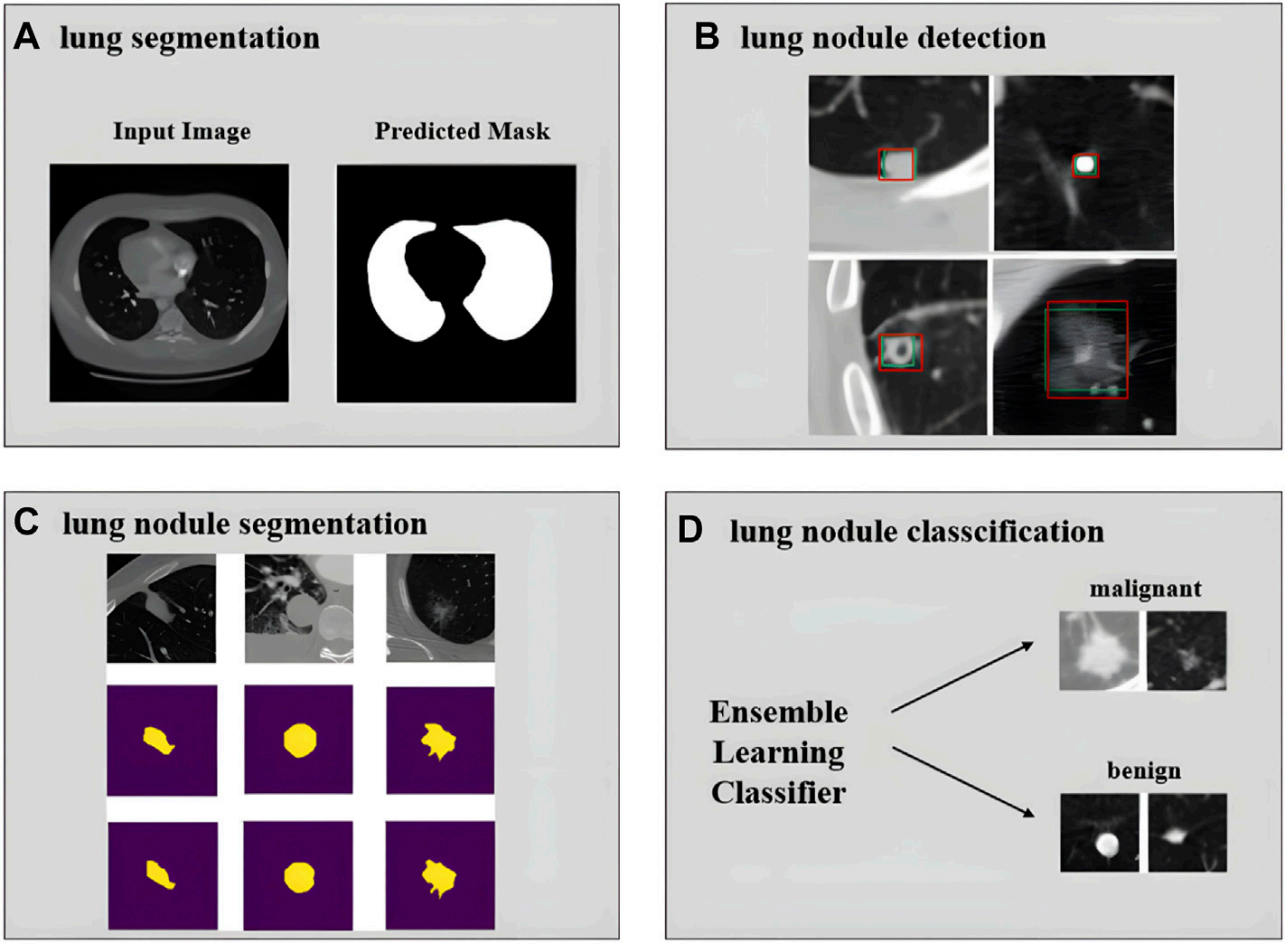
Achievements related to the realization of pulmonary nodule CAD system. **(A)** Input image and predicted mask for lung segmentation (adapted and modified from Tan et al., 2020). **(B)** Lung nodule detection results using deep learning. The green rectangle box represents ground truth and the red rectangle box represents the detection results (adapted and modified from Cao et al., 2019). **(C)** Segmentation results of large nodules, the first row is the original image, the second row is the radiologist’s manual annotation results, and the third row is the result of the network prediction (adapted and modified from Shi et al., 2020). **(D)** Classification of lung nodules into malignant and benign using an ensemble learning classifier (adapted and modified from Zhang et al., 2019).

Currently, many researchers focus on the development of a computer-aided diagnosis (CAD) system for pulmonary nodules on chest CT. The main processes are lung segmentation, lung nodule detection, lung nodule segmentation, lung nodule classification, etc. Tan et al. perform lung segmentation using a GAN network (Figure 9A) (Tan et al., 2021). Cao et al. implement lung nodule detection using a two-stage CNNs (Figure 9B) (Cao et al., 2020). Shi et al. use aggregation U-Net Generative Adversarial Networks for lung nodule segmentation (Figure 9C) (Shi et al., 2020). Zhang et al. use ensemble learners of multiple deep CNNs to classify lung nodules (Figure 9D) (Zhang et al., 2019). More experimental details also include feature extraction and false positive removal for lung nodules. The development of pulmonary nodule CAD system can help clinicians make diagnosis, reduce the workload of doctors, and has good application value and market prospect.

### 6.3 Optical coherence tomography combined with artificial intelligence

**FIGURE 10:**
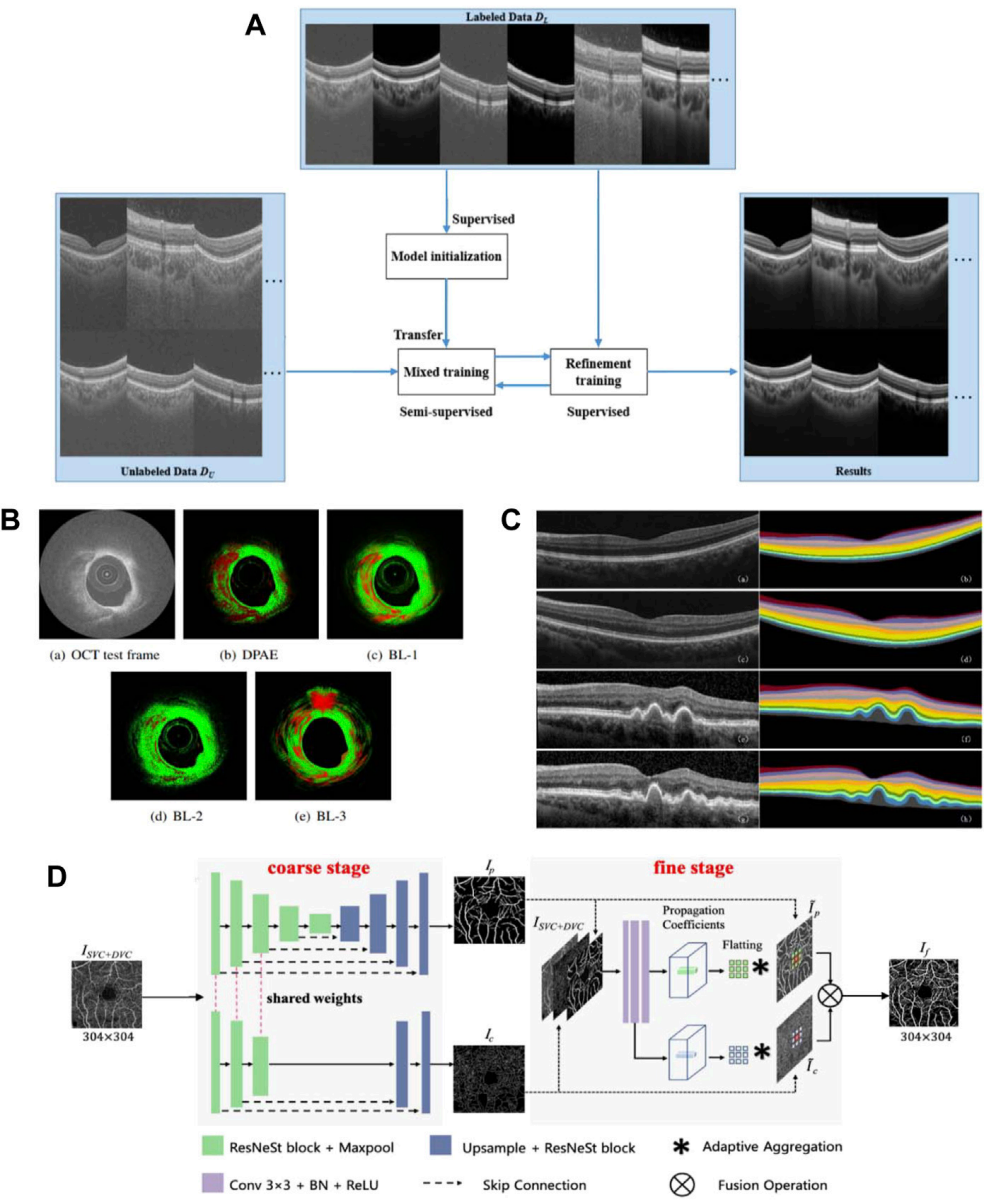
Related achievements of AI processing OCT images. **(A)** structure of the proposed semi-supervised system (adapted and modified from Wang et al., 2021). **(B)** The result of plaque detection, the red area represents the detected plaque, and the green area represents the normal tissue (adapted and modified from Roy et al., 2016). **(C)** Retinal 10-layer segmentation prediction results, The left is the original image, the right is the segmentation result (adapted and modified from Li et al., 2019). **(D)** Architecture of the proposed OCTA-Net network (adapted and modified from Ma et al., 2021).

Likewise, artificial intelligence has begun to develop in the field of OCT images. Since speckle noise can greatly affect the image quality of OCT, researchers seek to denoise images by using CNN and GAN. Wang et al. propose a semi-supervised learning method of GAN with fewer parameters to deal with the overfitting problem caused by too many parameters, and can use less data to complete the training (Figure 10A) (Wang et al., 2021b). Zhou et al. use CycleGAN to unify the style of images captured by different OCT instruments, and use conditional GAN for denoising (Zhou et al., 2022).

In the field of OCT intravascular imaging, researchers have applied artificial intelligence to assist in the diagnosis of atherosclerosis. Abhijit et al. propose a distribution preserving autoencoder based neural network for plaque detection in blood. To adapt to the spatiotemporal uncertainty of OCT speckle images, the model learns the representation in the data while preserving the statistical distribution of the data, and a newly proposed LogLoss function is used for error evaluation (Figure 10B) (Roy et al., 2016). To further identify vulnerable plaques, they propose to use a bag of random forests to learn tissue photon interactions (Roy et al., 2015). Asaoka et al. use deep learning to diagnose early-onset glaucoma based on macular OCT images, and use transfer learning to deal with differences in images acquired by different OCT devices (Asaoka et al., 2019). Thomas et al. construct a neural network of encoder and decoder, and complete the classification of fluid and non-fluid regions by semantic segmentation of OCT images, realizing the detection and quantification of macular fluid IRC and SRF, which is convenient for the diagnosis of exudative macular disease (Schlegl et al., 2018).

Research on automated segmentation of retinal OCT images contributes to the diagnosis of retinopathy-related diseases. Li et al. use an improved Xception65 to extract feature information, pass it into the spatial pyramid module to obtain multi-scale information, and finally used an encoder-decoder structure for retinal layer segmentation (Figure 10C) (Li et al., 2020b). Yang et al. achieve retinal layer segmentation with choroidal neovascularization, which responds to retinal morphological changes by introducing a self-attention mechanism (Yang et al., 2020). In (Roy et al., 2017), an end-to-end full CNN with encoder and decoder is constructed, realizing simultaneous segmentation of multiple retinal and fluid pockets to aid in the diagnosis of diabetic retinopathy. Artificial intelligence methods can also be combined with other methods for segmentation, Fang et al. conduct probability mapping on nine retinal layer boundaries through CNN and describe the boundary using the graph search method (Fang et al., 2017). In order to improve the segmentation accuracy, Srinivasan et al. first use sparsity-based image denoising, and then combine graph theory, dynamic programming and SVM to segment the ten-layer boundary of the mouse retina (Srinivasan et al., 2014).

Recently, researchers have begun to use optical coherence tomography angiography (OCTA) images to study retinal blood vessel segmentation. Compared with the more commonly used color fundus imaging techniques, OCTA can present subtle microvessels. Methods for vessel segmentation using deep learning can be roughly divided into two categories. The first category is to use multiple deep learning networks to refine the segmentation results. Ma et al. create a dataset ROSE containing 229 annotated OCTA images and propose a two-stage vessel segmentation network (OCTA-Net), where the coarse stage module is used to generate preliminary confidence maps, and the fine stage is to optimize vessel shape (Figure 10D) (Ma et al., 2021). The second is to enhance the ability of a single network to extract features. Mou et al. use U-Net as the basis and combined with self-attention mechanism to build a channel and spatial attention network, which can process various types of images from corneal confocal microscopy and OCTA (Mou et al., 2019). Li et al. propose an image projection network (IPN). The network architecture uses three-dimensional convolution and unidirectional pooling to achieve 3D-to-2D retinal vessel segmentation and foveal avascularzone segmentation (Li et al., 2020c). In (Liu et al., 2020b), unsupervised OCTA retinal vessel segmentation is proposed using encoders constructed from the same regions of different devices (Liu et al., 2020b).

### 6.4 Organoids combined with artificial intelligence

**FIGURE 11:**
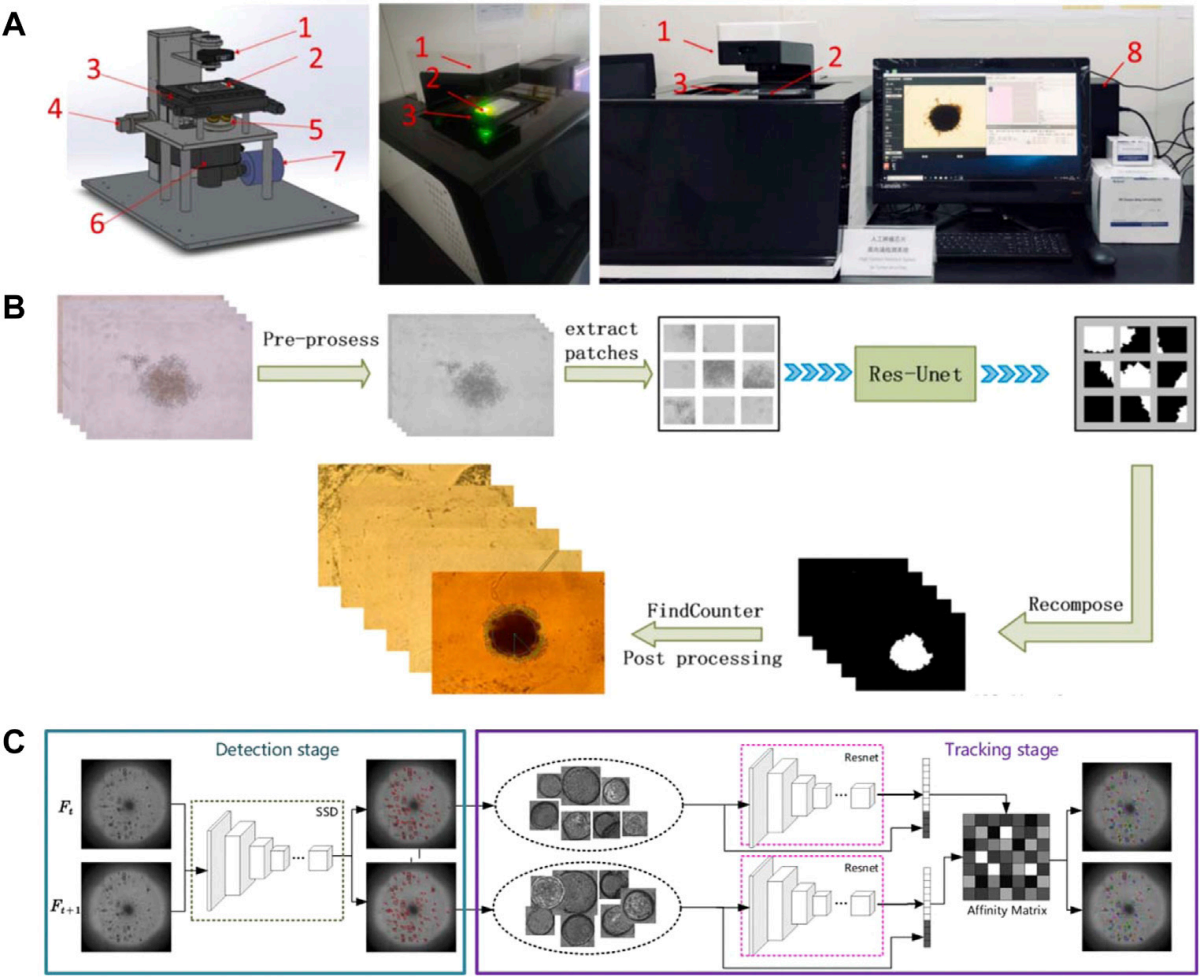
Relevant results of artificial intelligence combined with organoids. **(A–B)** System and process for edge detection of tumor spheres (adapted and modified from Chen et al., 2021b). **(A)** The SMART system for automated imaging and analysis. 1) Condenser with light source; 2) sample plate; 3) motorized x,y stage; 4) motorized Z-axis module; 5) objective wheel; 6) filter wheel; 7) CCD; 8) computer to control SMART system with developed software interface. **(B)** The process of tumor sphere edge detection. **(C)** Pipeline for organoids tracking (adapted and modified from Bian et al., 2021).

The development of artificial intelligence on OOC mainly focuses on the analysis of organoid images. Our team build a fully automated tumor spheres analysis system (Figure 11A) that integrates automatic identification, autofocus, and a CNN algorithm based on improved U-Net for accurate tumor boundary detection (Figure 11B). Moreover, two comprehensive parameters—the excess perimeter index and the multiscale entropy index are developed to analyze tumor invasion (Chen et al., 2021b). Bian et al. develop a deep learning model for detection and tracking of high-throughput organoid images. It is mainly implemented in two steps. The first step is to detect the organoids in the collected images of all periods, and the second step is to perform feature extraction on the detected organoids, and calculate the similarity of adjacent periods of organoids for tracking (Figure 11C) (Bian et al., 2021). Kegeles et al. use deep learning algorithms for retinal organoid differentiation, specifically using transfer learning to train a CNN for feature extraction and sample classification (Kegeles et al., 2020). Kong et al. use machine learning methods in colorectal and bladder organoid models to predict the efficacy of anti-cancer drugs in patients (Kong et al., 2020). In addition, researchers have improved deep learning methods for characterizing organoid models using augmented loss functions based on previous studies (Winkelmaier and Parvin, 2021). The development of organoids and OOC is unstoppable, and the application of artificial intelligence methods will undoubtedly bring greater vitality and impetus to the development of this field.

## 7 Discussion and conclusion

### 7.1 Discussion

1) A close combination between OOC or organoid and image-guided radiotherapy may provide extra benefits for the treatment of related diseases, especially in the field of oncology, which requires a more precise localization and efficient workflow. This challenge was overcome by a newly-designed integrated CT linear accelerator (linac) uRT-linac 506c, by achieving a diagnostic-quality visualization of anatomical structures and a seamless workflow (Yu et al., 2021). Artificial intelligence algorithm is also applied to the dose prediction of intensity-modulated radiotherapy plan generating to simply the clinical trial (Sun et al., 2022).
2) Medical imaging methods with high spatial resolution such as micro-CT, small animal MRI, and OCT are required for small-sized organoids. Some organoids with size reaching millimeter level and visible to the naked eye, such as tumor spheres, may be imaged by clinical MRI, and the instrument is easier to obtain.
3) In addition to the structural imaging mainly discussed in this article, positron emission tomography (PET) and MRS are very promising in combination with OOC to monitor biochemical changes in tissues. PET is often combined with CT or MRI. As the first total-body PET/CT scanner, the uEXPLORER can provide dynamic images with higher temporal resolution, sensitivity and signal-to-noise ratio, contributing higher feasibility to the proposed research (Marro et al., 2016; Cherry et al., 2018; Liu et al., 2021). Furthermore, benefited from the inherent advantages of MRI, PET/MR is expected to provide better soft tissue contrast compared to PET/CT. More promisingly, some previous researches show the higher sensitivity and specificity of integrated in the detection of micro lesions (Zhou et al., 2021). PET/CT and PET/MRI will provide multi-angle information for analyzing the changes and characteristics of tumor spheres.
4) In the future, as OOC enters the market, medical imaging instruments will be required to process multiple OOCs, most likely arrays of OOCs, simultaneously, and faster or even real-time imaging technology will be required. Exploring medical imaging instruments dedicated to OOC is both a challenge and an opportunity.
5) Artificial intelligence has been widely used in image analysis of medical imaging, including object detection, image segmentation, and image enhancement mentioned in this paper. In the same way, when medical imaging technology is used to image OOC, artificial intelligence will also support the development of OOC by automatically analyzing images.

### 7.2 Conclusion

Imaging of tissue-engineered artificial tissues and OOCs is in the ascendant. Admittedly, there are limited works utilizing medical imaging tools for tissue engineering and OOC researches. However, with the increase in the application of 3D tissue models and OOCs in drug discovery, environmental protection, and personalized medicine, we believe, in the very near future, the use of medical imaging technology to image micro-organs and use AI for analysis could be a mainstream methodology for organoid and OOC imaging. This paper reviews the research on medical imaging, artificial intelligence especially deep learning application and 3D tissue construction technology, as well as the combination of the two, which will provide relevant biomedical engineering researchers with effective imaging methods for different organoids, and lead to a more rapid development of research in this field.
